# Promoting Smoke-free Environments and Tobacco Cessation in Residential Treatment Facilities for Mental Health and Substance Addictions, Oregon, 2010

**Published:** 2011-12-15

**Authors:** Linda Drach, Daniel Morris, Cathryn Cushing, Cinzia Romoli, Richard Harris

**Affiliations:** Oregon Public Health Division, Program Design and Evaluation Services; Oregon Public Health Division and Oregon Addictions & Mental Health Division, Portland, Oregon; Oregon Public Health Division and Oregon Addictions & Mental Health Division, Portland, Oregon; Oregon Public Health Division and Oregon Addictions & Mental Health Division, Portland, Oregon; Oregon Public Health Division and Oregon Addictions & Mental Health Division, Portland, Oregon

## Abstract

We assessed tobacco-related policies and procedures at all state-funded, community-based residential mental health and substance addiction treatment facilities before implementation of new state policy requirements. We conducted telephone interviews with 162 of 166 (98%) facility administrators. Only 15% had voluntarily implemented 100% smoke-free campus policies, and 47% offered cessation resources at patient discharge; however, less than 10% expressed opposition to these future requirements. Smoking bans and cessation support in residential treatment facilities can reduce tobacco-related disparities among people with mental illness and addictions, but states may need to be the catalyst for policy implementation.

## Objective

Although the disproportionate tobacco use prevalence among people with mental illness and substance use disorders is well documented (1,2), few policies exist in the United States that address this problem. In Oregon, 3 statewide policy changes are under way at community-based residential mental health and addiction treatment facilities: 1) requiring 100% smoke-free campuses, 2) prohibiting staff from distributing tobacco products to residents, and 3) mandating integration of smoking cessation into discharge planning. We assessed current tobacco-related policies and procedures at all state-funded, mental health and drug addiction residential treatment facilities before policy implementation.

## Methods

In November 2010, Oregon's Addictions and Mental Health (AMH) and Public Health divisions partnered to collect baseline data from a census of state-funded, community-based residential treatment facilities for mental health and addiction (n = 166), to assess facility readiness to implement policy changes promoting tobacco cessation. Public health staff, with input from AMH partners, developed a brief survey that included 1 open-ended and 21 closed-ended items assessing policies and procedures related to indoor and outdoor smoking; evaluation of tobacco use at intake; promotion of cessation resources; and use of referrals, medications, and peer-based cessation support in residence and at discharge. This report focuses on the 3 statewide policy changes.

Two weeks before survey implementation, the AMH administrator sent a memorandum to treatment facility administrators, informing them of the upcoming survey and requesting their participation. The 166 facilities included 52 alcohol and drug treatment facilities (31.3%), 92 mental health treatment facilities (55.4%), and 22 secure mental health treatment facilities (13.3%). All provide 24-hour services. Secure facilities restrict a resident's exit by locking doors and gates. The average length of stay at Oregon residential facilities is approximately 100 days for alcohol and drug treatment and more than 1 year for mental health treatment (Oregon Health Authority, unpublished raw data, 2009).

Public Health staff conducted interviews by telephone with facility administrators or their designees. We analyzed data by using SPSS 17.0 (SPSS, Inc, Chicago, Illinois). We grouped brief answers from the open-ended item into broad themes by using content analysis (3). The project was classified as exempt from institutional review board review.

## Results

Ninety-eight percent of facilities completed surveys; 3 facilities could not be reached after multiple attempts, and 1 declined. Although all facilities reported indoor smoking bans, only 15% (n = 25) also prohibit outdoor smoking on all campus areas. Most (82%) reported existing policies prohibiting tobacco distribution by staff to residents, but alcohol and drug facilities (96%) were significantly more likely to have such restrictions than either secure (73%) or nonsecure mental health facilities (76%).

Approximately half of facilities (47%, n = 76) currently include follow-up referrals and cessation medications at discharge for residents who quit smoking in residence. These measures did not vary by facility type.

About 2 in 3 facility administrators (69%, n = 111) provided additional open-ended comments about tobacco-related policies. Most comments were neutral, but 2 sets of themes emerged from a subset of administrators. Administrators who favored smoke-free and cessation policies (n = 14) said that implementation and enforcement would be easier at the facility level with centralized leadership from the state:

"I'm pleased the state is going in this direction. . . . If we all go smoke-free at the same time, we can support each other and have less impact on the number of beds that are filled."

"It just needs to be done with everyone at once, at a state level. It's the right direction."

Administrators who opposed the policies (n = 10) cited residents' right to smoke and said smoking cessation is a lesser treatment priority:

"Forcing patients to stop smoking when there are so many other issues we have to deal with is ridiculous."

“I hope they never try to outlaw smoking [here] because they're adults ߞ not criminals ߞ and smoking is not a crime."

## Discussion

Thirty-five percent of Oregon adults who report "depression, anxiety, or emotional problems" smoke (4) compared with 16% overall (5); national rates among people with serious mental illness or addictions are higher (1,2). Adults with mental illness attempt to quit smoking at rates similar to others, but are less successful (6). Effective treatments for these populations are available (7), but concerns about interference with addiction treatment or exacerbation of psychiatric symptoms (8) have inhibited mental health and substance addiction facilities from offering cessation services to patients.

This assessment showed that few residential treatment facilities for mental health and substance addiction voluntarily implemented 100% smoke-free campuses, and only half mandated the integration of smoking cessation into discharge planning. However, fewer than 10% of administrators objected to these future tobacco policies, and about equal numbers welcomed such statewide policy changes.

Smoke-free campus and cessation policies can help residents quit smoking long-term (9). State-level policies, coupled with provider education and access to cessation supports, may be the necessary impetus for facilities to adopt evidence-based practices that can reduce illnesses and deaths among these patient populations.

Our study had several limitations. Although assured confidentiality, facility administrators may have overstated the presence of smoke-free policies. Also, strong written policies are not always demonstrated in daily practice; these data should not be assumed to reflect enforcement, compliance, or nonadministrative staff support.

There is a growing recognition that integration of tobacco cessation into mental health and substance addiction treatment is both urgently needed and possible (10). States can play a key role in ensuring that widespread policies addressing these tobacco-related disparities among people with mental health and substance addictions are adopted, implemented, and enforced.
