# Erratum, Vol. 9, Dec. 15 Release

**Published:** 2012-01-26

In the article “Promoting Smoke-free Environments and Tobacco Cessation in Residential Treatment Facilities for Mental Health and Substance Addictions, Oregon, 2010,” the suggested citation incorrectly identified the year of publication year as 2011. The correct year of publication is 2012. The correction was made to our website on January 19, 2012, and appears online at http://www.cdc.gov/pcd/issues/2012/11_0080.htm. We regret any confusion or inconvenience this error may have caused.

